# Nutritional Assessment of a Novel Processed Blend of Black Soldier Fly Larvae and Poultry By‐Product Meal as a Fishmeal Replacement in Nile Tilapia (*Oreochromis niloticus*) Diets

**DOI:** 10.1155/anu/9903025

**Published:** 2026-06-28

**Authors:** Eman Y. Mohammady, Ahmed M. Aboseif, Nasr M. Ahmed, Abdallah Tageldein Mansour, Roshmon Thomas Mathew, Mohamed Ashour

**Affiliations:** ^1^ Aquaculture Division, National Institute of Oceanography and Fisheries, NIOF, Cairo, Egypt, niof.sci.eg; ^2^ Freshwater and Lakes Division, National Institute of Oceanography and Fisheries, NIOF, Cairo, Egypt, niof.sci.eg; ^3^ Animal and Fish Production Department, College of Agricultural and Food Sciences, King Faisal University, Al-Ahsa, Saudi Arabia, kfu.edu.sa; ^4^ Fish Resources Research Center, King Faisal University, Hofuf-420, Al-Ahsa, 31982, Saudi Arabia, kfu.edu.sa

**Keywords:** black soldier fly larvae, enzymatic hydrolysis, *Hermetia illucens*, poultry by-product

## Abstract

As the world’s population rises, the aquaculture sector has put a lot of money into creating ecologically benign, long‐lasting protein sources to replace fishmeal (FM) in aquatic diets. This study evaluated the potential of applying a 1:1 mixture of processed black soldier fly larvae (BSL, *Hermetia illucens*) meal and poultry by‐product (PB) meal as a replacement for FM in the diets of Nile tilapia (*Oreochromis niloticus*). Both ingredients were treated with physical, chemical, and enzymatic hydrolysis treatments. A 70‐day feeding trial was conducted to investigate the effects of BSL–PB mixture on growth performance, digestive enzyme activities, feed utilization, nutrient digestibility, intestinal histomorphology, blood biochemical indicators, and hepatic antioxidant activities. Five experimental diets were created to replace FM with PB‐BSL at 0% (control), 25% (PB‐BSL_25%_), 50% (PB‐BSL_50%_), 75% (PB‐BSL_75%_), and 100% (PB‐BSL_100%_). A total of 450 Nile tilapia fry (3.78 ± 0.08 g) was stored in five groups, with three replicates per group. Each replicate was stocked in a 100 L fiberglass tank at a stock density of 30 fry/tank. During the 70‐day feeding period, fish were fed three times daily to ensure adequate nutrient intake. The fish were weighed once every 15 days throughout the experiment. Groundwater was used, and 10% of the water was replenished regularly. Results showed that groups of PB‐BSL_50%_ and PB‐BSL_75%_ (containing 50% and 75% PB‐BSL replacement) had significantly increased the values of growth performance, feed utilization, digestibility coefficients, digestive enzyme activity, intestinal histomorphometry, serum biochemical, and hepatic antioxidant activities compared to the control group. In contrast, groups receiving PB‐BSL_25%_ and PB‐BSL_100%_ [25% and 100% PB‐BSL replacement, respectively] replacement, respectively) had no negative effect on the overall health of Nile tilapia. Interestingly, the polynomial regression model for feed conversion ratio (FCR) and weight gain (WG) of *O. niloticus* indicated that replacing 50%–60% of FM with the processed PB‐BSL mixture provides an environmentally friendly and sustainable alternative to FM in Nile tilapia diets.

## 1. Introduction

The selection of components and the production of aquaculture diets significantly affect the environmental impact of the aquaculture industry [1]. Consequently, ongoing enhancement in this area is essential [2]. Exploring suitable and environmentally friendly substitutes for fishmeal (FM) and fish oil is a significant area of research, involving potential sources from plants, insects, animal by‐products, and algae [1, 3–5]. Processed animal proteins (PAPs) are derived from single‐gastric‐farmed animals suitable for human consumption at the final stage of slaughter [6–8]. PAPs have high levels of protein and amino acids and fewer carbohydrates than conventional animal food ingredients [9]. The diverse nutritional properties of animal by‐products, such as those from poultry processing, make them suitable as potential alternatives to FM in aquafeeds. Nonruminant PAPs are easily accessible and less expensive than FM, with an estimated 3–3.9 million tons produced annually in the EU [10]. Fish and crustacean diets already contain terrestrial animal by‐products, which might further lower FM incorporation levels in a more economical way [11]. Furthermore, the use of nonruminant PAPs can contribute to improving the sustainability and eco‐efficiency of aquaculture practices. Compared to conventional FM, this type of feed generally has a lower carbon footprint and provides an efficient way to utilize animal byproducts [12, 13].

Poultry by‐product (PB) meal is considered one of the most attractive sources of PAPs. It is a low‐cost, readily available, and nutrient‐rich protein source derived from rendered poultry by‐products. PB contains 450–650 g/kg protein and is high in essential amino acids (EAA), except methionine and lysine [9, 14–16]. However, there is growing interest in the use of black soldier fly larvae (BSL, *Hermetia illucens*) as a sustainable protein source for aquaculture diets [17–19]. BS has been considered a potential ingredient for aquatic animal diets because of its capacity to transform food waste (such as vegetables, fruit, and animal waste) into protein of superior quality [20]. BS has a high protein content (approximately 40%–65% of dry weight), an appropriate level of critical amino acids, and a high percentage of saturated fatty acids [21–23]. The nutritional value of BSL is affected by the processing method [24]. The defatted black soldier larvae (BSL) meal has a higher protein content compared to the full‐fat BSL meal [25]. In the BSL meal, the chitin content associated with the exoskeleton may negatively affect feed digestibility, with higher levels typically observed in the prepupal stage [26]. Chitin is considered a major factor that negatively affects the in vitro crude protein (CP) digestibility of BSL meal [27]. Smets et al. [28] reported that the amount of chitin increased in parallel with the development of *H. illucens*, being lowest at the larval stage (3.85%) and highest at the pupal stage (6.31%). The replacement of dietary FM with BSL meal has already been successfully tested in several aquatic species [29–33]. This study aims to evaluate the nutritional impacts of BSL meal and PB meal on Nile tilapia (*Oreochromis niloticus*) diets using a novel processing method, which includes physical, chemical, and enzymatic hydrolysis. This approach has not been previously studied.

## 2. Materials and Methods

### 2.1. Preparation of PB and BSL Meal

PB and BSL were treated with formic acid, as described by Fox et al. [34], with a few adjustments. In brief, the PB and BSL were autoclaved (10 min at 121°C under 2.09 kg/cm^2^) [35], then they were treated with formic acid and sun‐dried until the moisture content reduced to about 10%. About 100 g of dried PB and BSL were immersed in 300 mL of 3% formic acid at room temperature for 20 min. Then, they were filtered through a cheesecloth and rinsed with distilled water to raise the pH to 7. After being filtered, the solids were dried in the sun, crushed, and passed through a 1.0 mm mesh screen. Following that, the enzymatic hydrolysis procedure was carried out in the same method as reported by Uczay et al. [36], with some slight adjustments. Simply, 1 kg of each PB and BSL was thoroughly combined with 1 L of distilled water.

Then, a combined mixture of protease (P4860, Sigma–Aldrich Company), chitinase, and papain (P4760, Sigma–Aldrich Company) at a 1:1:1 (weight‐to‐weight) ratio was added. After incubation at a consistent temperature of 36°C/4 h. the enzymes were then inactivated by heating the hydrolyzed PB‐BSL mixture to 100°C for 10 min. The PB‐BSL were dried in a forced‐air oven at 55°C for 24 h for desiccation, and the samples are subsequently stored at −20°C until required. The proximate composition (protein, lipids, ash, fiber, and nitrogen‐free extract, NFE) of untreated/treated PB, BSL, and their mixture (PB‐BSL) was analyzed according to Feldsine et al. [37] (Table 1). Moreover, the amino acid contents of treated PB, BSL, their mixture (PB‐BSL) (Table 2), and the experimental diets (control diet, PB‐BSL_25%_, PB‐BSL_50%_, PB‐BSL_75%_, and PB‐BSL_100%_) (Table 3) were analyzed by an automated amino acid analyzer, following the protocol described by Kader et al. [39]

**Table 1:** Proximate composition (% dry matter) of poultry byproduct (BP), black soldier fly larvae meal (BSL), treated poultry byproduct meal, and their treated forms.

Parameter	Untreated PB	±SEM	Treated PB	±SEM	Untreated BSL	±SEM	Treated BSL	±SEM	Treated PB–BSL mixture	±SEM
Protein	30.62	0.63	30.75	0.76	42.50	0.68	42.68	0.12	37.82	0.45
Lipid	9.35	0.03	8.04	0.04	32.84	0.42	31.22	0.22	19.50	0.50
Nitrogen free extract	33.64	0.95	47.37	0.34	3.86	0.54	13.41	0.82	30.52	0.60
Crude Fiber	21.91	0.32	10.23	0.51	10.58	0.44	5.34	0.61	6.82	0.40
Ash	4.48	0.34	3.61	0.25	10.22	0.32	7.35	0.45	5.34	0.30

*Note:* The values are presented as means ± standard error of triplicate groups (*n* = 3). Data are presented for descriptive purposes only; no statistical comparisons (*p*‐values) were performed among the ingredients. Nitrogen‐free extract = 100 − (crude protein + lipid + ash + fiber content). PB‐BSL, a treated mixture of PB and BSL.

Abbreviations: BSL, black soldier fly larvae; PB, poultry byproduct.

**Table 2:** Amino acid profile (% dry matter) of treated poultry byproduct (PB), black soldier fly larvae (BSL), and their mixture (PB‐BSL).

Amino acid	Amino acid composition		
	Treated PB	Treated BSL	PB‐BSL mixture
Essential amino acids (EAA)			
Arginine	2.42	3.42	3.00
Histidine	1.00	1.94	1.98
Lysine	2.58	3.85	3.64
Methionine	0.90	1.25	1.09
Leucine	3.42	4.32	3.90
Isoleucine	1.71	2.24	1.99
Threonine	1.70	2.50	2.30
Phenylalanine	1.92	2.36	2.25
Valine	2.00	3.81	2.70
Total EAA	17.65	25.69	22.85
Nonessential amino acids (NEAA)			
Glutamic acid	7.42	6.51	6.85
Aspartate acid	3.41	5.92	4.56
Serine	2.30	2.43	2.75
Glycine	2.52	3.49	3.09
Alanine	2.14	3.78	2.69
Tyrosine	1.34	3.80	2.46
Total NEAA	19.13	25.93	22.40
Total amino acids (TAA)	36.78	51.62	45.25

*Note:* BSL: black soldier fly larvae meal; their mixture PB‐BSL: a treated mixture of PB and BSL.

Abbreviation: PB, poultry byproduct.

**Table 3:** Composition and proximate analysis of experimental diets (g/kg dry matter).

Ingredients	Diets				
	Control	PB‐BSL_25%_	PB‐BSL_50%_	PB‐BSL_75%_	PB‐BSL_100%_
Fishmeal	100	75	50	25	0
Treated PB–BSL mixture	0	42.2	84.5	126.7	169
Soybean meal	380	380	380	380	380
Corn gluten	70	70	70	70	70
Yellow corn	240	240	240	240	240
Wheat bran	150	132.8	115.5	98.3	81
Soybean oil	40	40	40	40	40
Vitamin and mineral mixture^a^	20	20	20	20	20
Proximate analysis					
Crude protein	312.0	310.5	309.1	307.6	306.2
Crude lipid	68.3	73.0	77.8	82.6	87.4
Ash	54.3	50.9	47.6	44.2	40.9
Crude fiber	52.9	52.8	52.7	52.6	52.5
NFE	512.5	512.8	512.8	513.0	513.0
Gross energy (MJ/kg diet)^b^	18.86	19.02	19.17	19.33	19.49
Essential amino acids					
Threonine	12.5	12.4	12.3	12.1	12.0
Phenylalanine	13.0	12.9	12.7	12.5	12.3
Isoleucine	13.1	12.9	12.7	12.5	12.3
Leucine	24.0	23.8	23.5	23.2	22.9
Valine	14.5	14.4	14.2	14.0	13.8
Histidine	5.3	5.3	5.4	5.5	5.6
Lysine	20.5	19.5	19.0	18.4	17.8
Arginine	16.5	16.4	16.2	16.0	15.8
Methionine	7.2	7.1	7.0	6.8	6.7
Tryptophan	3.5	3.4	3.3	3.2	3.1
Total essential amino acids	130.1	128.1	126.3	124.2	122.3
Nonessential amino acids					
Serine	23.0	23.0	22.0	21.0	20.0
Glutamic	65.4	64.2	63.4	62.6	61.8
Glycine	22.9	22.6	21.8	20.1	19.3
Alanine	30.1	30.1	29.3	28.7	27.1
Tyrosine	12.4	12.4	11.8	11.6	11.4
Proline	24.0	24.4	23.8	22.2	21.6
Cystine	5.2	5.7	5.8	5.9	6.0
Aspartic	50.0	50.0	49.1	48.1	47.0
Total nonessential amino acids	233.0	232.4	227.0	220.2	214.2
Total amino acids	363.1	360.5	353.3	344.4	336.5

*Note:* Control: Diet with 100% fishmeal, PB‐BSL_25%_, PB‐BSL_50%_, PB‐BSL_75%_, and PB‐BSL_100%_: Diets are mixture of treated PB‐BSL as a replacing of fishmeal at levels of 25% (PB‐BSL_25%_), 50% (PB‐BSL_50%_), 75% (PB‐BSL_75%_), and 100% (PB‐BSL_100%_), respectively. PBS: A treated mixture of poultry byproducts (PB) and black soldier larvae (BSL). Fishmeal was obtained from a commercial supplier, poultry by‐product meal was sourced from a local rendering plant, and black soldier fly larvae were provided from a local breeder and subsequently processed into meal. Soybean meal, yellow corn, corn gluten, wheat bran, and soybean oil were purchased from the local market.

^a^Vitamin and mineral mixture (g/kg) of mixture contains: 4800 I.U. Vit A, 2400 IU cholecalciferol (Vit. D), 40 g Vit E, 8 g Vit K, 4.0 g Vit B12, 4.0 g Vit B2, 6 g Vit B6, 4.0 g, pantothenic acid, 8.0 g nicotinic acid, 400 mg folic acid, 20 mg biotin, 200 g choline, 4 g copper, 0.4 g iodine, 12 g iron, 22 g manganese, 22 g zinc, 0.04 g selenium, folic acid, 1.2 mg; niacin, 12 mg; d‐calcium pantothenate, 26 mg; pyridoxine. HCl, 6 mg; riboflavin, 7.2 mg; thiamin. HCl, 1.2 mg; sodium chloride (NaCl, 39% Na, 61% Cl), 3077 mg; ferrous sulfate (FeSO_4_·7H_2_O, 20% Fe), 65 mg; manganese sulfate (MnSO_4_, 36% Mn), 89 mg; zinc sulfate (ZnSO_4_·7H_2_O, 40% Zn), 150 mg; copper sulfate (CuSO_4_·5H_2_O, 25% Cu), 28 mg; potassium iodide (KI, 24% K, 76% I).

^b^Gross energy calculated using gross calorific values of 23.63, 39.52, and 17.15 kJ/g for protein, fat, and carbohydrate, respectively according to Brett [38].

### 2.2. Diet Formulation

Five practical diets were formulated to be isonitrogenous and approximately isoenergetic (Table 3) to meet the dietary requirements of Nile tilapia [40]. The control diet contains 10% FM. In the PB‐BSL diets, FM protein was partially or fully replaced with a mixture of processed PB‐BSL (a blend of BSL and PB meal) at 25% (PB‐BSL_25%_), 50% (PB‐BSL_50%_), 75% (PB‐BSL_75%_), and 100% (PB‐BSL_100%_). A pellet mill (San Francisco, CA, USA) was used to produce pellets after the dry ingredients and soybean oil were combined for 5 min. After drying at ambient temperature, the pellets were stored at 4°C. The Feldsine et al. [37] methods were used to determine the gross energy and proximate analysis of diets (Table 3).

### 2.3. Fish Husbandry

All experiment methods were conducted following the regulations and guidelines approved by the National Institute of Oceanography and Fisheries (NIOF) Committee for Institutional Care of Aquatic Organisms and Experimental Animals (NIOF‐AQ4‐F‐25‐R‐023). All procedures followed the ARRIVE guidelines (https://arriveguidelines.org). The monosex *O. niloticus* came from a commercial farm close to Kafr El‐Sheekh, Government of Egypt. Fish were given the control diet at a level of 3% of total biomass in equal portions three times a day (9 a.m., 11 a.m., and 15 p.m.) during the 15‐day acclimation period at the fish nutrition laboratory of NIOF. The fish’s initial body weight was 3.78 ± 0.08 g. After acclimation, 450 samples were divided into five groups, each with three replicates, and placed in 100 L fiberglass tanks at a density of 30 fish per tank. During the experiment, groundwater was used, and 10% of the water was replenished regularly. The oxygen requirements were satisfied by compressed air. The fish were fed three times a day for 70 days and weighed every 2 weeks during the experiment. The American Public Health Association (APHA) [41] standard procedures were used to record the values of temperature, pH, dissolved oxygen, ammonia, nitrate, and nitrite of the ambient water. Water quality parameters were tested daily, and temperature values (26.2–28.2°C) were within the acceptable range for Nile tilapia growth. Dissolved oxygen levels were maintained at 6.0 ± 0.03 mg/L, while ammonia was 0.15 ± 0.02 mg/L. Nitrite (NO_2_) concentrations averaged 0.02 ± 0.004 mg/L, and nitrate (NO_3_) levels were 0.74 ± 0.06 mg/L [42].

### 2.4. Growth and Feed Utilization

At the end of the experiment, fish were gently removed from each tank and immediately weighed to minimize handling stress without the use of anesthesia to determine their final weight gain (WG). The feed utilization and growth performance indicators were investigated using the formulas referenced in the footnote to Table 4.

**Table 4:** Growth and feed utilization of Nile tilapia *O. niloticus* fed the experimental diets for 70 days.

Parameter	Diets					±SEM	*p*‐Value
	Control	PB‐BSL_25%_	PB‐BSL_50%_	PB‐BSL_75%_	PB‐BSL_100%_		
IBW (g/fish)	3.80	3.89	3.82	3.72	3.65	0.03	0.435
WG (g/fish)	19.81^bc^	20.29^b^	21.30^a^	21.26^a^	18.53^c^	1.45	0.044
SGR (% day)	2.61^b^	2.59^b^	2.69^a^	2.72^a^	2.58^b^	0.08	0.001
FI (g/fish)	35.94	34.20	34.41	32.60	33.98	1.56	0.142
FCR	1.81^a^	1.68^b^	1.61^c^	1.53^d^	1.83^a^	0.12	0.031
PER	1.77^c^	1.91^b^	2.00^a^	2.12^a^	1.78^d^	0.18	0.027

*Note:* Means followed by different superscript letters in the same row are significantly different (*p* < 0.05, one‐way ANOVA). Abbreviations and formulas: control = diet with 100% fishmeal; PB‐BSL_25%_, PB‐BSL_50%_, PB‐BSL_75%_, and PB‐BSL_100%_ = diets in which fishmeal protein was replaced with a treated mixture of poultry byproduct (PB) and black soldier fly larvae (BSL) at 25%, 50%, 75%, and 100%, respectively. Weight gain (WG, g) = final weight (FW, g) – initial body weight (IBW, g); specific growth rate (SGR, % day^−1^) = [Ln(W2) – Ln(W1)]/*t* × 100, where W1 and W2 are initial and final body weights (g), and *t* = duration of the experiment (days, 70 days); feed intake (FI, g/fish) = total feed consumed per fish; feed conversion ratio (FCR) = FI/WG; protein efficiency ratio (PER) = WG/protein intake (g).

### 2.5. Determination of Endogenous Intestinal Digestive Enzymes

At the end of the experiment, four fish per replicate were dissected after fish were anesthetized by 3‐aminobenzoic acid ethyl ester at 100 mg/L (MS‐222, Sigma–Aldrich, St. Louis, MO, USA). Then, the intestine samples were promptly homogenized in 10 volumes (w/v) of ice‐cold physiological saline solution, and the supernatant was then kept for endogenous enzyme activity measurement [43]. Amylase and lipase activities were measured using the methods established by Bernfeld [44] and Zamani et al. [45], respectively. Based on the method conducted by Hummel [46], the activities of chymotrypsin and trypsin were assessed.

### 2.6. Digestibility

5 g/kg food chromic oxide (Cr_2_O_3_) was used as an external indicator to calculate the diets’ apparent digestibility coefficients (ADCs) [47]. From each tank, 10 fish were transferred to fiber aquariums to collect excrement consistently. Each tank was cleared of any remaining food after 2 h of feeding. Feces were collected by siphoning through a 0.2‐mm fine‐mesh net [48]. The biochemical assessment was determined according to Feldsine et al. [37]. Nutrient digestibility was determined using the formulas of Schneider et al. [49].

### 2.7. Intestinal Histomorphometry Examination

After the feeding trial, midsections of intestines from three fish per replicate were dissected following anesthesia with 3‐aminobenzoic acid ethyl ester (MS‐222; 100 mg/L; Sigma–Aldrich, St. Louis, MO, USA), and the intestinal samples were collected. The samples were then rinsed with phosphate‐buffered saline (PBS) to remove any adhering contents, kept in 10% formalin for 24 h, dehydrated in an escalating series of alcohols, and cleaned in xylene. Subsequently, paraffin wax (melting point: 58°C–60°C) was used to cover the samples. A rotatory microtome (Reichert Technologies, NY, USA) was used to cut longitudinal and transverse slices, each 6 μm thick. Hematoxylin and eosin (H&E) staining was performed in accordance with the recommended technique [50]. A light microscope (Olympus BX‐50) combined with image analysis software (Leica Microsystems, Germany) was used to examine the tissue. Image analysis software was used to measure the average villus height and the absorption surface area (from the base to tip) for statistical performance.

### 2.8. Blood Sampling and Analysis

From each group, three samples were randomly chosen, sedated with MS 222 (100 mg/L, Sigma, St. Louis, MO), and blood samples were obtained from the caudal vein. A method that was described by Reitman and Frankel [51] was utilized in order to evaluate the activity of liver enzymes, such as aspartate aminotransferase (AST) and alanine aminotransferase (ALT), by utilizing the serum that was obtained from the blood samples. Henry [52] and Wotton and Freeman [53] were used to figure out the total serum protein and albumin levels. To identify globulin, albumin was deducted from the total protein.

### 2.9. Hepatic Antioxidants Measurements

In each replicate, three fish were anesthetized with 3‐aminobenzoic acid ethyl ester (MS‐222; 100 mg/L; Sigma–Aldrich, St. Louis, MO, USA), after which their livers were randomly collected, weighed, washed, and homogenized. The homogenates were then mixed with ice‐cold saline at a 1:9 ratio (0.1 g liver to 0.9 mL of saline, pH 7.0). The homogenates were centrifuged, and the supernatants were used for biochemical analyses. Superoxide dismutase (SOD) was determined according to Peskin and Winterbourn [54], malondialdehyde (MDA) according to Ohkawa et al. [55], catalase (CAT) following Beers and Sizer [56], glutathione (GSH) according to Rodriguez‐Ariza et al. [57], and glutathione peroxidase (GPx) according to Nagai et al. [58].

### 2.10. Statistical Analysis

**Figure 1:**
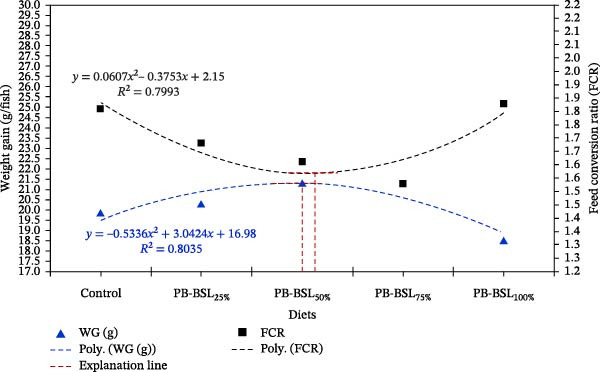
Polynomial regression chart between feed conversion rate (FCR) and weight gain (WG) of Nile tilapia *O. niloticus* fed the experimental diets for 70 days. Control: diet with 100% fishmeal, PB‐BSL_25%_, PB‐BSL_50%_, PB‐BSL_75%_, and PB‐BSL_100%_: diets in which fishmeal protein was replaced with a treated mixture of PB‐BSL at 25%, 50%, 75%, and 100%, respectively. PB‐BSL: A treated mixture of poultry byproducts (PB) meal and black solider fly larvae meal (BSL).

Before starting data analysis, consistency and uniformity tests were performed. The data were analyzed using one‐way ANOVA, utilizing SAS software [59] (version 6.03, Soft Inc., USA). The mean differences were evaluated using Duncan’s multiple‐range test (*p*  < 0.05). The data were presented as means with standard errors of the mean (±SEM). The polynomial regression assessment model (Figure 1) was conducted in Excel.

## 3. Results

### 3.1. Proximate Composition of PB Meal, BSL Meal, and Their Mixture (PB‐BSL)

Table 1 shows the proximate composition of untreated and treated PB and BSL as well as their mixture (PB‐BSL). BSL, both untreated and treated, had the highest protein and lipid contents compared to PB and the PB‐BSL mixture. In contrast, crude fiber was the highest in untreated PB, followed by untreated BSL and treated PB, with the lowest levels observed in treated BSL. The PB–BSL mixture showed intermediate values for protein, lipid, NFE, fiber, and ash compared with the individual treated PB or BSL samples. Table 2 reports that the EAA were found to be higher in the treated BSL (25.69%) than in the treated PB (17.65%). In contrast, the nonessential amino acids (NEAA) were found to be higher in the treated BSL (25.93%) than in the treated PB (19.13%). The total amino acids were 51.62% and 45.25% in treated BSL and blend PB‐BSL, respectively (Table 2).

### 3.2. Growth and Feed Utilization Indices

Table 4 shows the growth and feed utilization of *O. niloticus* fed the experimental diets for 70 days. Compared with the control diet, *O. niloticus* growth and nutrient utilization were significantly affected by the inclusion of PB‐BSL. WG, specific growth rate (SGR), and protein efficiency ratio (PER) were significantly higher at PB‐BSL_50%_ and PB‐BSL_75%_ groups compared to the control group (*p* ≤ 0.05), while the lowest values were observed in PBS_100%_ group. Feed intake (FI) didn’t differ significantly among the experimental groups (*p*  > 0.05). Feed conversion ratio (FCR) was lowest in the PB‐BSL_75%_ group, followed by PB‐BSL_50%_ (*p*  < 0.05). In summary, fish performance was improved with partial PB‐BSL replacement (50%–75%), whereas the total replacement of FM with PB‐BSL (100%) had an adverse effect.

Figure 1 shows the polynomial regression analysis between FCR and WG in the fish *O. niloticus*. According to the obtained data, the regression for WG increased (*R* ^2^ = 0.7993), and the regression for FCR significantly decreased (*R* ^2^ = 0.8035) at the PB‐BSL50% replacement level. The broken explanation line determines that the ideal FM replacement level is between 50% and 60% based on these findings. This range closely matches the PB‐BSL_50%_ group, which showed the best balance between feed and growth efficiency, with the FCR reaching its lowest value and WG reaching its highest peak.

### 3.3. Intestinal Endogenous Digestive Enzymes Activity

The digestive enzyme activities of Nile tilapia fed the experimental diets for 70 days are shown in Table 5. Fish fed the PB‐BSL_75%_ diet showed the greatest chymotrypsin activity, while those fed on the PB‐BSL_50%_ diet exhibited the highest trypsin activity. Lipase activity was significantly influenced by PB‐BSL levels (*p* ≤ 0.05), with the highest activity observed in PB‐BSL_50%_ and PB‐BSL_75%_ (1082 U/g and 1080 U/g). Amylase activity also increased significantly (*p* ≤ 0.05) with PB‐BSL inclusion, reaching a maximum at 75% PB‐BSL (744 U/g). Digestive enzyme activities were improved when FM was partially substituted with PB‐BSL, especially at PB‐BSL_50%_.

**Table 5:** Digestive enzymes of Nile tilapia *O. niloticus* fed the experimental diets for 70 days.

Enzymes (U/g tissue)	Diets					±SEM	*p*‐Value
	Control	PB‐BSL_25%_	PB‐BSL_50%_	PB‐BSL_75%_	PB‐BSL_100%_		
Chymotrypsin	7.42	7.72	8.21	8.43	7.05	1.26	0.068
Trypsin	3.42	3.54	4.41	4.01	3.11	0.50	0.071
Lipase	985^b^	1015^ab^	1082^a^	1080^a^	988^b^	6.66	0.005
Amylase	647^d^	694^c^	718^b^	744^a^	650^d^	7.29	0.004

*Note:* Means followed by different superscript letters in the same row are significantly different (*p* < 0.05, one‐way ANOVA). Control = diet with 100% fishmeal; PB‐BSL_25%_, PB‐BSL_50%_, PB‐BSL_75%_, and PB‐BSL_100%_ = diets in which fishmeal protein was replaced with a treated mixture of poultry byproduct (PB) and black soldier fly larvae (BSL) at 25%, 50%, 75%, and 100%, respectively. PB‐BSL, a treated mixture of poultry byproduct (PB) and black soldier larvae (BSL).

### 3.4. ADCs

Table 6 demonstrates the ADCs (%) of Nile tilapia *O. niloticus* fed the experimental diets for 70 days. No significant (*p*  > 0.05) differences in the ADCs of dry matter (DM), CP, and crude lipid (CL) for fish fed PB‐BSL inclusion levels. Moderate inclusion of treated PB‐BSL (PB‐BSL_50%_–PB‐BSL_75%_) improved CP and lipid digestibility compared with the control and other treatments, whereas full replacement (PB‐BSL_100%_) reduced them. DM digestibility remained consistent across all treatments with no significant differences (*p*  > 0.05). Overall, partial replacement of FM with PB‐BSL (50%–75%) enhanced nutrient digestibility, while full replacement (100%) had a negative effect.

**Table 6:** Apparent digestibility coefficients (%) of Nile tilapia *O. niloticus* fed the experimental diets for 70 days.

Nutrient parameter	Diets					±SEM	*p*‐Value
	Control	PB‐BSL _25%_	PB‐BSL _50%_	PB‐BSL _75%_	PB‐BSL _100%_		
Dry matter	74.29	74.51	73.95	74.00	74.32	0.55	0.9035
Crude protein	83.70	85.44	86.42	87.60	80.80	0.93	0.6215
Crude lipid	86.51	86.35	89.97	90.43	80.96	0.76	0.4216

*Note:* Values are means ± SEM (*n* = 3). No significant differences (*p*  > 0.05, one‐way ANOVA) were observed among the dietary treatments. Control = diet with 100% fishmeal; PB‐BSL_25%_, PB‐BSL_50%_, PB‐BSL_75%_, and PB‐BSL_100%_ = diets in which fishmeal protein was replaced with a treated mixture of poultry byproduct (PB) and black soldier fly larvae (BSL) at 25%, 50%, 75%, and 100%, respectively. PB‐BSL, a treated mixture of poultry byproduct (PB) and black soldier larvae (BSL).

### 3.5. Intestinal Histomorphometry

**Figure 2:**
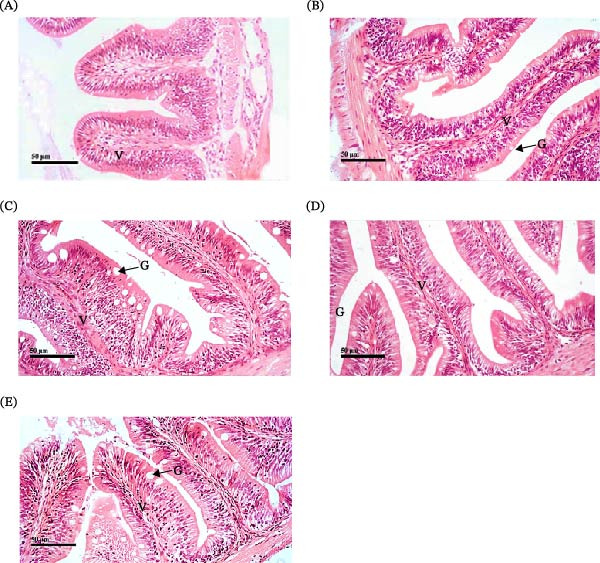
Histoarchitecture of small intestine sections of Nile tilapia stained with hematoxylin&eosin (H&E) showing the branching anastomosing mucosal villi (V) with goblet cells (G). (A) The control diet. (B) A mixture of treated poultry byproduct meal and treated Black solider fly larvae (PB‐BSL) as a substitute of FM at level of 25% (PB‐BSL_25%_). (C) A mixture of treated poultry byproduct meal and treated black solider fly larvae (PB‐BSL) as a substitute of FM at level of 50% (PB‐BSL_50%_). (D) A mixture of treated poultry byproduct meal and treated black solider fly larvae (PB‐BSL) as a substitute of FM at level of 75% (PB‐BSL_75%_). (E) A mixture of treated poultry byproduct meal and treated black solider fly larvae (PB‐BSL) as a substitute of FM at level of 100% (PB‐BSL_100%_). (H&E. × 400): scale bar = 50 μm.

Table 7 and Figure 2 illustrate the histomorphometry of Nile tilapia *O. niloticus* fed the experimental diets for 70 days. Histomorphometric analysis showed significant differences in intestinal structure among treatments (*p*  < 0.05). Compared with the control group (Figure 2A), the mucosal layer was transformed into long, finger‐like villi that extended into the intestinal lumen and were covered with simple columnar epithelial cells known as enterocytes (Figure 2B–E). Based on the results, the PB‐BSL_50%_ – PB‐BSL_75%_ diets showed the most favorable effects on intestinal structure, particularly for villi width and goblet cell numbers, with no significant difference between them. The PB‐BSL_25%_ – PB‐BSL_50%_ treatments recorded the highest values for villi height and muscularis mucosa, also without significant differences between them (Figure 2C,D). However, full replacement (PB‐BSL_100%_) led to a decline in these parameters, indicating a negative impact on intestinal morphology at excessive levels (Figure 2E).

**Table 7:** Histomorphometry of the intestine of Nile tilapia, *O. niloticus* fed the experimental diets for 70 days.

Parameter	Experimental treatments					±SEM	*p*‐Value
	Control	PB‐BSL _25%_	PB‐BSL _50%_	PB‐BSL _75%_	PB‐BSL _100%_		
Villi width (mm)	211.50^c^	221.62^b^	242.21^a^	244.00^a^	210.14^c^	2.71	0.005
Villi height (mm)	436.00^b^	439.31^a^	445.71^a^	437.02^b^	239.33^c^	5.11	0.008
Goblet cells (No.)	17.60^b^	16.54^c^	18.08^a^	18.90^a^	16.52^c^	1.23	0.015
Muscularis mucosa (mm)	42.87^b^	43.08^a^	43.86^a^	44.39^a^	42.00^c^	1.27	0.028
Height muscularis (mm)	219.30^c^	228.45^b^	235.13^a^	233.95^a^	200.28^d^	4.71	0.001

*Note:* Means followed by different superscript letters in the same row are significantly different (*p*  < 0.05, one‐way ANOVA). Control = diet with 100% fishmeal; PB‐BSL_25%_, PB‐BSL_50%_, PB‐BSL_75%_, and PB‐BSL_100%_ = diets in which fishmeal protein was replaced with a treated mixture of poultry byproduct (PB) and black soldier fly larvae (BSL) at 25%, 50%, 75%, and 100%, respectively. PB‐BSL, a treated mixture of poultry byproduct (PB) meal and black soldier larvae (BSL).

### 3.6. Serum Biochemical Indices

Table 8 displays the serum biochemical profile of Nile tilapia *O. niloticus* fed the experimental diets for 70 days. Inclusion of PB‐BSL significantly affected serum biochemical parameters (*p* ≤ 0.05). Moderate inclusion of PB‐BSL_50%_ – PB‐BSL_75%_ reduced ALT and AST levels and increased total protein, albumin, and globulin concentrations. Total FM replacement with PB‐BSL led to a decline in these serum biochemical markers.

**Table 8:** Serum biochemical indices of Nile tilapia, *Oreochromis niloticus* fed the experimental diets for 70 days.

Biochemical parameters	Experimental treatments					±SEM	*p*‐Value
	Control	PB‐BSL _25%_	PB‐BSL _50%_	PB‐BSL _75%_	PB‐BSL _100%_		
ALT(U/L)	17.02^a^	13.62^c^	12.03^d^	11.97^d^	15.45^b^	2.82	0.015
AST(U/L)	89.25^a^	83.31^b^	79.41^c^	78.02^d^	87.13^a^	3.21	0.048
Total protein (g/dL)	2.65^c^	3.54^b^	3.84^a^	3.92^a^	2.52^c^	1.25	0.005
Albumin (g/dL)	1.57^c^	1.62^b^	1.76^ab^	1.89^a^	1.50^c^	1.70	0.019
Globulin (g/dL)	1.08^c^	1.92^b^	2.08^a^	2.03^a^	1.02^c^	3.65	0.001

*Note:* Means followed by different superscript letters in the same row are significantly different (*p*  < 0.05, one‐way ANOVA). Control = diet with 100% fishmeal; PB‐BSL_25%_, PB‐BSL_50%_, PB‐BSL_75%_, and PB‐BSL_100%_ = diets in which fishmeal protein was replaced with a treated mixture of poultry byproduct (PB) and black soldier fly larvae (BSL) at 25%, 50%, 75%, and 100%, respectively. PB‐BSL, a treated mixture of poultry byproduct (PB) meal and black soldier larvae (BSL).

Abbreviations: ALT, alanine aminotransferase; AST, aspartate aminotransferase.

### 3.7. Hepatic Antioxidant Activities

**Figure 3:**
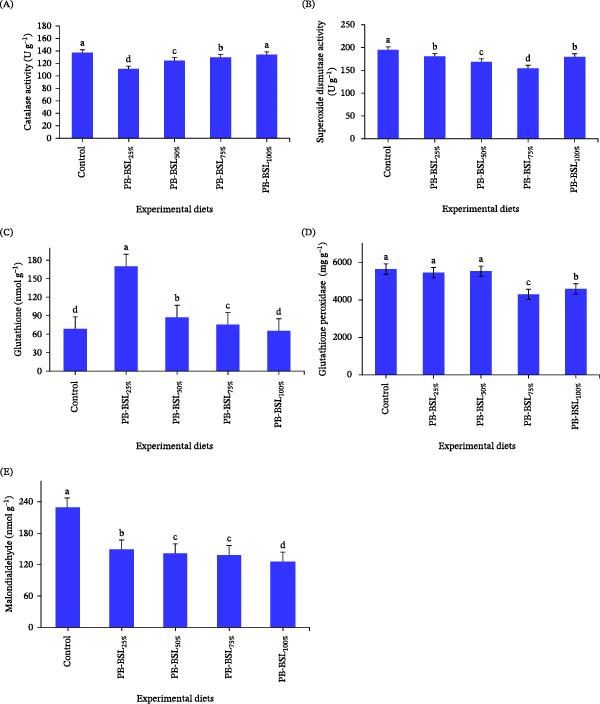
(A–E): Antioxidant enzymes activities of Nile tilapia fed different experimental diets for 70 days; (A) catalase (CAT) u g^−1^; (B) superoxide dismutase (SOD) u g^−1^; (C) glutathione (GSH) nmol g^−1^; (D) glutathione peroxidase (GPx); and (E) malondialdehyde (MDA) nmol/g. Different letters in columns indicate significant differences among treatments (*p*  < 0.05).

Figure 3 shows the activities of hepatic antioxidant enzymes of Nile tilapia *O. niloticus* fed the experimental diets for 70 days. The inclusion of PB‐BSL in the diet significantly affected liver antioxidant enzyme activity in fish (*p* ≤ 0.05). Moderate PB‐BSL levels (PB‐BSL_25%_ – PB‐BSL_75%_) significantly increased GSH, while GPx activity was not significantly affected at these inclusion levels compared with the control (Figures 3C,D). In contrast, CAT and SOD were significantly higher (*p* ≤ 0.05) than in other treatments (Figure 3A,B). MDA levels were reduced in all PB‐BSL‐fed groups, with the lowest value observed in the PB‐BSL_100%_ group (Figure 3E).

## 4. Discussion

In aquaculture feeds, environmental and sustainable substitutes for FM have emerged as one of the most critical issues in aquatic organisms [60, 61]. The hypothesis of this study is that a mixture of PB meal and BSL, processed through multistep methods including autoclaving, acid treatment, and enzymatic hydrolysis, may synergistically enhance the growth performance of Nile tilapia, enabling effective FM replacement. The BSL‐PB blend was specifically designed to provide complementary nutritional benefits to the two main ingredients. Rather than increasing the quantity of protein, which consists mainly of BSL’s very high CP content, aiming to improve the quality of proteins for Nile tilapia. PB protein has a moderate protein content relative to theCP of BSL; however, the protein from PB is deficient in EAAs, primarily methionine and lysine; therefore, the relative EAA content in a BSL–PB mixture will be approximately balanced to compensate for the low levels of the EAAs in PB. BSL balances these drawbacks with a more balanced amino acid profile. Thus, the PB‐BSL mixture has both sufficient protein content and increased total amino acids and a better EAA profile that means a higher protein quality source for Nile tilapia feed. The treatment increases the digestibility and feed value of the whole mixture. The variable chitin content of BSL is supported by the high‐protein content of PB. The amino acid profile of BSL (rich in methionine and lysine) [62, 63] and its lipid content compensate for this deficiency in PB. To our knowledge, this study is the first to evaluate the combination of PB and BSL in this way. This study aims to reduce the dependence on FM in the Nile tilapia diet by partially or fully replacing it with a processed BSL larvae and PB meal. The results of this study provide strong evidence that a combination of BSL and PB successfully serves as a sustainable feed substitute when processed through multiple steps (autoclaving, acid treatment, and enzymatic hydrolysis), with significant advantages for Nile tilapia performance. This novel formulation has not been explored. Results showed that partial substitution of FM with treated PB‐BSL blend at 50% and 75% levels (PB‐BSL_50%_ and PB‐BSL_75%_) significantly improved WG, PER, and SGR. This improvement is probably due to the enhancements of the nutrient profile, reduction of antinutritional factors (ANF), and changes in the digestibility and bioavailability of nutrients due to processing. These benefits are related to diets with a proper balance of the EAAs, especially lysine, methionine, and arginine, which are indispensable for protein synthesis and growth. The better PER and lower FCR observed were because the PB‐BSL diets contained their adequate inclusion level. The total replacement of FM at PB‐BSL_100%_ could have led to a deficiency of some bioactive compounds or EAAs naturally present in FM, which may explain the reduced growth parameters and protein utilization at this level, had less balanced amino acid profiles or higher replacement of FM, resulting in lower growth performance and feed efficiency. The pattern highlights the significance of the total protein content and the quality and balance of amino acids in diets as major determinants of growth and nutrient utilization in Nile tilapia. The PB‐BSL blend offered a more nutritionally balanced feed by complementing the PB protein with the high amino acid profile of BSL, thus favoring optimal growth at moderate inclusion levels. These results are in accordance with previous studies, which showed that insect‐based meals can successfully replace FM partially without affecting the performance [64]. These results highlight the potential of processed BSL and PB as sustainable feed ingredients, particularly when used at partial replacement levels, to support growth and feed efficiency in Nile tilapia while reducing reliance on traditional FM. In this study, acid hydrolysis treatment with formic acid was chosen for hydrolysis since it was previously reported to improve the nutritional quality and in vitro digestibility of shrimp head meal, a chitin‐rich feed, more than NaOH, HCl, and autoclaving [65]. In addition, in vivo studies in broiler chickens have shown improved growth performance and nutrient digestibility when formic acid‐treated shrimp meal is included [66]. Formic acid and its salts are among the organic acids used as feed additives to enhance fish health and performance [67]. Moreover, enzymatic hydrolysis is a promising biotechnology application that can reduce ANFs and enhance the bioavailability of essential protein sources [68]. The findings are consistent with several earlier studies that the enzymatic hydrolysis improved the nutritional quality and functional properties of Nile tilapia *O. niloticus* proteins [69, 70], whiteleg shrimp, *Litopenaeus vannamei* [71], and Japanese flounder, *Paralichthys olivaceus* [72]. These enzymatically hydrolyzed amino acids play a crucial role in enhancing the nutritional quality and palatability of aquafeed [73]. Our findings are consistent with many previous works that report substituting FM with BSL fly larvae or PB meals, at various levels, has a significant impact on growth performances and feed utilization in different aquatic animal models [74–78].

However, our data indicate that a processed PB‐BSL blend can be a promising alternative to FM, with substitution levels ranging from 50% to 75%. This could be attributed to the use of multiprocessing in the PB‐BSL blend, which likely reduced fiber, chitin, and ANFs, making it easier for Nile tilapia to digest. In another study, Mahmoud et al. [79] found that the replacement of FM protein with PB meal at different levels (50%, 75%, and 100%) in Nile tilapia diets significantly improved the growth and feed utilization efficiency (FW, WG, SGR, and PER) of fish. Our findings are in contrast with those of Tippayadara et al. [80], who reported no significant (*p*  > 0.05) improvement in growth or feed efficiency of Nile tilapia when replacing FM with BSL meal at varying levels (0% to 100%) compared to FM diet. Furthermore, there was a reduction in the growth in Nile tilapia fed diets containing formic acid‐treated BSL [81]. Consistent with current findings, partial FM substitution (up to 8.89%) with enzymatically hydrolyzed PB enhanced the growth efficiency of largemouth bass (*Micropterus salmoides*), but higher replacement levels negatively affected the final body weight and feed utilization [82]. In contrast, the partial replacement of FM with enzyme‐digested PB meal treatments exhibited growth responses of juvenile red drum (*Sciaenops ocellatus*) similar to those of the basal diet [83]. These differences could be due to several factors like fish species, size, and the specific formulation of the diets. However, the enzymatic hydrolysis converted a portion of insoluble protein into water‐soluble peptides and free amino acids, increasing protein solubility and enhancing digestibility in feed ingredients (e.g., protein hydrolysates from bone‐rich PBs) compared to untreated proteins [84].

The activity of endogenous digestive enzymes is essential for nutrient utilization and growth as they are responsible for the breakdown of dietary components and their subsequent assimilation [85]. In our study, partial replacement of FM with PB‐BSL (mainly at 50%–75%) resulted in increased digestive enzyme activities, suggesting better digestion of lipids and carbohydrates. The increased activities of digestive enzymes were observed, which could be due to the processing of the PB‐BSL mixture. The mixture was initially hydrolyzed with formic acid and then with a mixture of exogenous enzymes, including papain, protease, and chitinase. This process lowers the chitin content and other ANFs while making protein easier to digest and absorbing more nutrients. Also, when proteins break down during hydrolysis, they produce small peptides that help the intestine absorb nutrients and regulate digestive enzymes [86–88]. The increase in amylase activity may also be explained by the changed carbohydrate composition of the hydrolysates, which can improve the carbohydrate digestion efficiency [89]. Overall, these mechanisms enhanced the digestibility of proteins and bioactive peptides and appear to synergistically improve lipase and amylase activities, thus improving the utilization of lipids and carbohydrates. Another important reason for the increased digestive enzymes may be closely related to the increased intestinal histomorphology. As shown in the present data, these groups had significantly higher villi height and width, increased goblet cell numbers and greater muscularis thickness (*p* ≤ 0.05), which in turn indicate an increased absorptive surface area and better intestinal functionality. These structural improvements may promote more efficient nutrient digestion and absorption, which stimulates digestive enzyme secretion. On the contrary, the reduction of these histological parameters in PB‐BSL_100%_ may justify the reduction of the digestive efficiency at the highest inclusion levels. As far as is known, no studies have been carried out on the effect of treated PB–BSL mixture on the digestive enzyme activities in Nile tilapia.

Dawood et al. [90] reported that replacing FM with *Saccharomyces cerevisiae*‐fermented PB (FPBM) at 10% or 20% in common carp (*Cyprinus carpio*) diets improved the growth, digestive enzyme activities, feed efficiency, and immune and antioxidant responses. Similarly, Dawood et al. [91] reported that the dietary low inclusion of FPBM 11.17%–25.14% enhanced the WG and SGR of Nile tilapia (*O. niloticus*) and lower FCR, whereas their higher inclusion (40%) led to significant reduction of digestive enzymes, growth performance, and feed utilization. Using a polynomial regression model, our results indicate that the optimal replacement level for FCR and WG of *O. niloticus* is 50% to 60%. The differences between those previous results and our results may be attributed to the technique used in yeast‐fermented PB meal and to our processing technique (autoclaving, acid treatment, and enzymatic hydrolysis) in a PB‐BSL mixture.

Digestibility has a strong role in the absorption and utilization of fish nutrients [92]. Numerically, the ADCs for CP and CL were slightly higher in fish fed PB‐BSL_50%_ and PB‐BSL_75%_ diets compared with the control and other treatments; however, these differences weren’t statistically significant (*p*  > 0.05). Overall, the inclusion of PB‐BSL had no significant (*p*  > 0.05) effect on feed digestibility in Nile tilapia. Chitin is a structural component of crustacean sources such as shrimp meal and insect‐based sources such as shrimp meal and BSL that can reduce digestibility, and its processing may have decreased its content and enhanced the overall digestibility [27, 93].

Fox et al. [34] reported that growth and survival of *Penaeus monodon* were improved when fed diets containing formic acid‐treated shrimp head meal (SM). In support of this, Kim and Patterson [94] demonstrated that enzyme‐ and NaOH‐treated feather meal from dead hens exhibited increased pepsin digestibility without substantially altering its chemical composition. Our results are on the same line with the results reported by Soliman et al. [95], who found that there were no significant (*p*  > 0.05) differences in ADC of DM, CP, and NFE content among different diets containing PB (0%, 50%, and 100%) rather than FM in the Nile tilapia’s diet. On the other hand, Muin and Taufek [78] reported that the ADCs for DM, energy, and CP were significantly (*p* ≤ 0.05) higher in hybrid red tilapia fed BSL fly larvae meal compared to those fed FM in their diets. In the study of Perez‐Velazquez et al. [81], treating black soldier fly meal with formic acid had little effect on its elemental nutritional composition and did not improve the digestibility of CP and DM in Nile tilapia. Treated shrimp head meal, a chitin‐rich feed hydrolyzed with formic acid, exhibited improved digestibility and nutrient absorption in broilers [66].

Reda et al. [96] reported that the diet of tilapia containing dietary organic acids, including formic acid, improves digestion, enhances nutrient and mineral absorption, strengthens resistance to bacterial infections, and supports better growth. Similarly, formic acid inclusion in trout’s (*Oncorhynchus mykiss*) diet significantly improved the absorption of key minerals like phosphorus, magnesium, and calcium [97]. Belghit et al. [75] observed that replacing FM and soy protein concentrate with BSL in Atlantic salmon *Salmo salar* diets resulted in a reduction of the ADC of protein, lipid, and all amino acids. Several fish species, including meager, *Argyrosomus regius* [77], juvenile turbot, *Psetta maxima* [74], and sea bream, *Pagrus major* [98], have shown low ADC when fed regimes containing BSL meal. This reduction was mainly attributed to the higher chitin content found in insect meal‐based feeds [99]. However, as reported by several studies, the species’ abilities to digest chitin are varied [26, 100, 101]. In some cases, chitin is recognized for its immunostimulatory properties, providing protection against infections and diseases [102–104].

On the same line, the current results differ from those reported by Palupi et al. [76], who observed significantly lower ADCs for all nutrients when PB meal replaced FM at levels of 5%–30% (*p* ≤ 0.05). In contrast, the ADCs of protein and lipid in the PB‐BSL groups (up to PB‐BSL_75%_) in the present study weren’t significantly different from the control (*p*  > 0.05), indicating that partial replacement with the processed PB‐BSL mixture did not adversely affect nutrient digestibility in Nile tilapia. Similarly, Poolsawat et al. [105] reported that diets containing feather meal (FeM) led to reduced growth performance and lower ADCs in tilapia (*O. niloticus* × *O. aureus*), which they attributed to poor digestibility and an unbalanced amino acid profile of FeM. These findings highlight that the impact of alternative protein sources on digestibility depends on the type of the ingredient, its processing, and inclusion level. In contrast, Mendoza et al. [106] found that treating FeM with protease enzymes improved its nutritional value due to the fact that the enzymatic process breakdown of the keratin into smaller forms could be more easily absorbed by Pacific white shrimp *L. vannamei*.

In aquatic species, the intestine is essential for nutrient absorption and digestion [107]. Its barrier activity is crucial for preserving intestinal efficiency [108]. Several authors concluded that the intestinal histomorphological parameters, including the number of goblet cells, villi height, villi width, and the thickness of the muscular layer, are among the leading indicators controlling fish’s capacity to absorb nutrients and digestion, which in turn affects feed utilization and growth performance [109]. Goblet cells can help protect the intestinal mucosa through mucin secretion from dehydration, physical damage, and invading pathogens through mucin secretion [110, 111]. Based on the results, the PB‐BSL_50%_ – PB‐BSL_75%_ diets showed the most favorable effects on intestinal structure, particularly for villi width and goblet cell numbers, with no significant difference between them. The PB‐BSL_25%_–PB‐BSL_50%_ treatments recorded the highest values for villi height and muscularis mucosa, also without significant differences between them. Overall, both PB‐BSL_50%_ and PB‐BSL_75%_ diets demonstrated the most notable improvements in the intestinal structure compared with the other treatments. However, PB‐BSL_100%_ led to a decline in these parameters, indicating a negative impact on intestinal morphology at excessive levels. The negative impact of increasing the FM substituent with PB‐BSL could be attributed to the high chitin content in BSL. These findings were previously supported by Yao et al. [112], in which replacing FM with the BSL in the diet of the Chinese mitten crab, *Eriocheir sinensis*, led to improved intestinal morphology at 25%–50% replacement levels. These findings are also consistent with the results reported by Xu et al. [113], who observed similar effects when replacing FM with BSL in the diet of juvenile mirror carp (*C. carpio* var. specularis).

Hematological markers are essential indicators of the overall health and stress levels of aquaculture species. These indicators are effective tools for evaluating the effects of different formulated diets [114]. Our outcomes showed that, compared with the control treatment, moderate levels (PB‐BSL_50%_ and PB‐BSL_75%_) improved the liver function and protein metabolism in Nile tilapia. It may be attributed to the better absorption and digestion of smaller molecules in a processed blend, which could improve the fish’s metabolism. Despite full FM replacement with (PB‐BSL_100%_), hematological markers declined, suggesting potential negative effects on the fish’s liver. The current findings indicate that hematological markers were improved when FM was replaced with BSL‐PB at levels of 25%–75%. In the same direction, Yu et al. [115] observed that incorporating 8.89% enzymatically hydrolyzed PB into the diet resulted in a significant enhancement in blood markers of largemouth bass (*M. salmoides*). The current findings contrast with Zhou et al. [116], who recorded that replacing FM with BSL meal at levels of 35–140 g/kg had no effect on biochemical indicators in Jian carp *C. carpio*. Similarly, Yildirim‐Aksoy et al. [117] found that hybrid tilapia, Nile × Mozambique (*Oreocromis niloticus* × *O. mozambique*) fed diets containing 30% BSL meal showed no effects on hematological parameters. Eid et al. [118] reported that Nile tilapia (*O. niloticus*) fed diets containing different levels of fermented PB meal (0%, 25%, 50%, and 75%) did not show any negative impact on blood biochemical parameters or liver and kidney functions. Also, FM can be wholly replaced by either PB meal or fermenting PBM with baker’s yeast *S. cerevisiae* and *Lactobacillus casei* at 0%, 33.33%, 66.67%, and 100% levels in GIFT tilapia diets without adverse effects on hematological parameters [119]. Abdel‐Tawwab et al. [120] found that there were no significant changes in white blood cell counts, lymphocytes, monocytes, and neutrophils between European sea bass, *Dicentrarchus labrax*, fed BSL meal and those fed FM. However, the differences between our results and those of previous studies may be due to the specific processing techniques used to produce the PB‐BSL mixture (autoclaving, acid treatment, and enzymatic hydrolysis), as well as the differences in the methods used in those studies.

In aquatic animals, the hepatic antioxidant enzyme activities, including CAT, SOD, GSH, and GPx, play an essential role in improving aquatic animals’ health and protecting the liver from oxidative stress [121, 122]. The current results indicated that PB‐BSL inclusion affected antioxidant status, with increased GSH levels at PB‐BSL_25%_, PB‐BSL_50%_, and PB‐BSL_75%_, while GPx showed no significant differences among these levels. However, moderate‐to‐high PB‐BSL levels (PB‐BSL _25%_–PB‐BSL_75%_) generally enhance antioxidant enzyme activity and reduce lipid peroxidation. However, the PB‐BSL_100%_ level is detrimental as it reduces enzyme activities (SOD and GPx). However, MDA decreased across all treatments. Overall, more favorable antioxidant responses were observed at lower to intermediate inclusion levels, which is consistent with previous findings of Dawood et al. [91] who found that dietary inclusion of fermented PB meal at 30% improved GPx without affecting the MDA level in Nile tilapia, indicating improved antioxidant activity. These results disagree with the results investigated by Yao et al. [112], who reported that the replacement of FM with BSL meal at 25%–75% did not differ significantly on the antioxidant enzyme activities in juvenile Chinese mitten crab (*Eriocheir sinensis*), while the highest level, 100% replacement, resulted in the lowest activities of antioxidant enzymes and the highest MDA content, suggesting increased oxidative stress. The contradiction between our results and the other literatures may be due to differences in the preparation methods of the PB‐BSL mixture, including processing technique (autoclaving, acid treatment, and enzymatic hydrolysis). Additionally, it may be attributed to the fish type or experimental conditions.

## 5. Conclusion

The current study concluded that a multistep processed mixture of BSL meal and PB meal can effectively replace FM in Nile tilapia diets without any harmful effects on Nile tilapia’s growth, health, or overall performance evaluation. The combination of autoclaving, acid treatment, and the enzymatic hydrolysis process significantly improved the nutritional quality and digestibility of treated PB and BSL. According to the polynomial regression model, our results indicate that the optimal replacement level for FCR and WG of *O. niloticus* is 50% to 60%. Overall, this substitution of FM with the PB–BSL mixture offers an eco‐friendly alternative for tilapia production. Future work should focus on long‐term trials and the economic feasibility of large‐scale application across aquaculture species.

## Author Contributions

**Eman Y. Mohammady**: conceptualization, methodology, visualization, investigation, software, writing – review and editing, writing – original draft preparation, investigation. **Ahmed M. Aboseif**: writing – review and editing, writing – original draft preparation. **Nasr M. Ahmed**: methodology, software, writing – original draft preparation.**Abdallah Tageldein Mansour**: visualization, software, writing – review and editing, writing – original draft preparation, investigation. **Mohamed Ashour**: visualization, investigation, software, writing – review and editing, writing – original draft preparation, investigation.

## Funding

This work was supported by the Deanship of Scientific Research, Vice Presidency for Graduate Studies and Scientific Research, King Faisal University, Saudi Arabia (Grant KFU260206).

## Disclosure

All authors have approved the final version of the manuscript. After using Grammarly AI tool, the authors thoroughly reviewed, edited, and verified the entire content manually and take full responsibility for the scientific integrity and accuracy of the publication.

## Ethics Statement

All experiment methods in this study were carried out in accordance with the relevant guidelines and regulations. The experimental protocols were approved by the National Institute of Oceanography and Fisheries (NIOF) Committee for Institutional Care of Aquatic Organisms and Experimental Animals (NIOF‐AQ4‐F‐25‐R‐023). All procedures followed the ARRIVE guidelines (https://arriveguidelines.org).

## Conflicts of Interest

The authors declare no conflicts of interest.

## Data Availability

The datasets used and/or analyzed during the current study are available from the corresponding author upon reasonable request.

## References

[1] Boyd C. E. and McNevin A. A. , Front Matter, Aquaculture, Resource Use, and the Environment, 2015, John Wiley & Sons, Inc, i–xxv.

[2] Belghita I. , Liland N. S. , and Gjesdal P. , et al.Black Soldier Fly Larvae Meal Can Replace Fish Meal in Diets of Sea-Water Phase Atlantic Salmon (*Salmo salar*), Aquaculture. (2019) 503, 609–619, 10.1016/j.aquaculture.2018.12.032.

[3] Wan A. H. L. , Davies S. J. , Soler-Vila A. , Fitzgerald R. , and Johnson M. P. , Macroalgae as a Sustainable Aquafeed Ingredient, Reviews in Aquaculture. (2018) 11, 458–492.

[4] Fantatto R. R. , Mota J. , and Ligeiro C. , et al.Exploring Sustainable Alternatives in Aquaculture Feeding: The Role of Insects, Aquaculture Reports. (2024) 37, 10.1016/j.aqrep.2024.102228, 102228.

[5] Rosle S. , Mohd Rahim M. S. , Agustono A. , and Hassin N. H. , Alternative Feeds for Sustainable Aquaculture: A Comprehensive Structured Review, Journal of Science, Technology and Innovation Policy. (2024) 10, no. 2, 1–11, 10.11113/jostip.v10n2.150.

[6] Woodgate S. L. , Wan A. H. L. , Hartnett F. , Wilkinson R. G. , and Davies S. J. , The Utilisation of European Processed Animal Proteins as Safe, Sustainable and Circular Ingredients for Global Aquafeeds, Reviews in Aquaculture. (2022) 14, no. 3, 1572–1596, 10.1111/raq.12663.

[7] Woodgate S. L. , Meat Industry By-Products: A Bio-Refinery Approach to the Production of Safe, Value Added Products for Sustainable Agriculture Applications, Frontiers in Animal Science. (2023) 4, 10.3389/fanim.2023.1259200, 1259200.

[8] European Commission , Commission Regulation (EC) No 142/2011, 2011, Official Journal of the European Union, 1–254, L54.

[9] Karapanagiotidis I. T. , Psofakis P. , Mente E. , Malandrakis E. , and Golomazou E. , Effect of Fishmeal Replacement by Poultry By-Product Meal on Growth Performance, Proximate Composition, Digestive Enzyme Activity, Haematological Parameters and Gene Expression of Gilthead Seabream (*Sparus aurata*), Aquaculture Nutrition. (2019) 25, no. 1, 3–14, 10.1111/anu.12824.

[10] EFPRA/Fastmarkets , Animal Protein Market Coverage: Launch of New Price Assessments – Animal Protein Market Size and Production in Europe, 2025, European Fat Processors and Renderers Association (EFPRA). Online Industry report https://www.fastmarkets.com/insights/animal-protein-market-coverage-launch-of-new-price-assessment.

[11] Luthada-Raswiswi R. , Mukaratirwa S. , and O’Brien G. , Animal Protein Sources as a Substitute for Fishmeal in Aquaculture Diets: A Systematic Review and Meta-Analysis, Applied Sciences. (2021) 11, no. 9, 10.3390/app11093854, 3854.

[12] den Hartog L. A. and Sijtsma S. R. , Sustainable Feed Ingredients, *12th International Symposium of Australian Renderers Association “Rendering for Sustainability”*, 2013, Victoria, Australia, July 23-26, 2013.

[13] Zhang Z. , Liu H. , Jin J. , Zhu X. , Han D. , and Xie S. , Towards a Low-Carbon Footprint: Current Status and Prospects for Aquaculture, Water Biology and Security. (2024) 3, no. 4, 10.1016/j.watbs.2024.100290, 100290.

[14] Yaseen M. A. , Iqbal W. , and Bhatti S. A. , et al.Dietary Supplementation of Protease and Organic Acid in Poultry By-Product Meal-Based Diet in Broilers, Animal Bioscience. (2024) 37, no. 12, 2145–2154, 10.5713/ab.24.0136. 38938025 PMC11541022

[15] González-Rodríguez A. , Celada J. D. , Carral J. M. , Sáez-Royuela M. , García V. , and Fuertes J. B. , Evaluation of Poultry By-Product Meal as Partial Replacement of Fish Meal in Practical Diets for Juvenile Tench (Tinca tinca L.), Aquaculture Research. (2016) 47, 1612–1621.

[16] Zhou Q. C. , Zhao J. , Li P. , Wang H. L. , and Wang L. G. , Evaluation of Poultry By- Product Meal in Commercial Diets for Juvenile Cobia (Rachycentron canadum), Aquaculture. (2011) 322, 322–323.

[17] Oteri M. , Di Rosa A. R. , Lo Presti V. , Giarratana F. , Toscano G. , and Chiofalo B. , Black Soldier Fly Larvae Meal as Alternative to Fish Meal for Aquaculture Feed, Sustainability. (2021) 13, no. 10, 10.3390/su13105447, 5447.

[18] Eide L. H. , Rocha S. D. , and Morales-Lange B. , et al.Black Soldier Fly Larvae (*Hermetia illucens*) Meal Is a Viable Protein Source for Atlantic Salmon (*Salmo salar*) During a Large-Scale Controlled Field Trial Under Commercial-Like Conditions, Aquaculture. (2024) 579, 10.1016/j.aquaculture.2023.740194, 740194.

[19] Radhakrishnan G. , Philip A. J. P. , and Caimi C. , et al.Evaluating the Fillet Quality and Sensory Characteristics of Atlantic Salmon (*Salmo salar*) Fed Black Soldier Fly Larvae Meal for Whole Production Cycle in Sea Cages, Aquaculture Reports. (2024) 35, 10.1016/j.aqrep.2024.101966, 101966.

[20] Nairuti R. N. , Musyoka S. N. , Yegon M. J. , and Opiyo M. A. , Utilization of Black Soldier Fly (*Hermetia illucens Linnaeus*) Larvae as a Protein Source for Fish Feed: A Review, Aquaculture Studies. (2021) 22, no. 2, 10.4194/AQUAST697, AQUAST697.

[21] Shumo M. , Osuga I. M. , and Khamis F. M. , et al.The Nutritive Value of Black Soldier Fly Larvae Reared on Common Organic Waste Streams in Kenya, Scientific Reports. (2019) 9, no. 1, 1–13, 10.1038/s41598-019-46603-z. 31300713 PMC6626136

[22] Cattaneo A. , Meneguz M. , and Dabbou S. , The Fatty Acid Composition of Black Soldier Fly Larvae: The Influence of Feed Substrate and Applications in the Feed Industry, Journal of Insects as Food and Feed. (2023) 10, no. 4, 533–558, 10.1163/23524588-20230068.

[23] Sangsawang A. , Kovitvadhi S. , and Pewhom A. , et al.Impacts of Substituting Fish Meal With Full-Fat or Defatted Black Soldier Fly (*Hermetia illucens*) Larvae on Growth, Quality, and Health of Nile Tilapia (*Oreochromis niloticus*) Fingerlings, Aquaculture Reports. (2024) 38, 10.1016/j.aqrep.2024.102348, 102348.

[24] Zulkifli N. F. N. M. , Seok-Kian A. Y. , Seng L. L. , Mustafa S. , Kim Y.-S. , and Shapawi R. , Nutritional Value of Black Soldier Fly (*Hermetia illucens*) Larvae Processed by Different Methods, PLoS One. (2022) 17, no. 2, 10.1371/journal.pone.0263924, e0263924. 35213590 PMC8880436

[25] Saputra I. and Lee Y. , Nutrition Composition of Commercial Full-Fat and Defatted Black Soldier Fly Larvae Meal (*Hermetia illucens*) as a Potential Protein Resource for Aquafeeds, Biodiversitas Journal of Biological Diversity. (2023) 24, no. 9, 4877–4884, 10.13057/biodiv/d240930.

[26] Eggink K. M. , Pedersen P. B. , Lund I. , and Dalsgaard J. , Chitin Digestibility and Intestinal Exochitinase Activity in Nile Tilapia and Rainbow Trout Fed Different Black Soldier Fly Larvae Meal Size Fractions, Aquaculture Research. (2022) 53, no. 16, 5536–5546, 10.1111/are.16035.

[27] Bonomini M. G. , Prandi B. , and Caligiani A. , Black Soldier Fly (*Hermetia illucens* L.) Whole and Fractionated Larvae: In Vitro Protein Digestibility and Effect of Lipid and Chitin Removal, Food Research International. (2024) 196, 10.1016/j.foodres.2024.115102, 115102. 39614512

[28] Smets R. , Verbinnen B. , Van De Voorde I. , Aerts G. , Claes J. , and Van Der Borght M. , Sequential Extraction and Characterisation of Lipids, Proteins, and Chitin From Black Soldier Fly (Hermetia illucens) Larvae, Prepupae, and Pupae, Waste Biomass Valoriz. (2020) 11, 6455–6466.

[29] Hossain M. S. , Fawole F. J. , Labh S. N. , Small B. C. , Overturf K. , and Kumar V. , Insect Meal Inclusion as a Novel Feed Ingredient in Soy-Based Diets Improves Performance of Rainbow Trout (*Oncorhynchus mykiss*), Aquaculture. (2021) 544, 10.1016/j.aquaculture.2021.737096, 737096.

[30] He Y. , Liu X. , and Zhang N. , et al.Replacement of Commercial Feed With Fresh Black Soldier Fly (*Hermetia illucens*) Larvae in Pacific White Shrimp (*Litopenaeus vannamei*), Aquaculture Nutrition. (2022) 2022, no. 1, 10.1155/2022/9130400, 9130400.

[31] Limbu S. M. , Shoko A. P. , and Ulotu E. E. , et al.Black Soldier Fly (*Hermetia illucens*, L.) Larvae Meal Improves Growth Performance, Feed Efficiency and Economic Returns of Nile Tilapia (*Oreochromis niloticus*, L.) Fry, Aquaculture, Fish and Fisheries. (2022) 2, no. 3, 167–178, 10.1002/aff2.48.

[32] Maranga B. , Kagali R. , Mbogo K. , Orina P. , Munguti J. , and Ogello E. , Growth Performance of African Catfish (*Clarias Gariepinus*) Fed on Diets Containing Black Soldier Fly (*Hermetia Illucens*) Larvae Under Aquaponic System, Aquaculture Studies. (2022) 23, no. 5, 10.4194/AQUAST910, AQUAST910.

[33] Yakti W. , Shaw C. , Müller M. , Mewis I. , Kloas W. , and Ulrichs C. , Tracing the Journey of Elements from Fish Feed to Nile Tilapia Faeces to Black Soldier Fly Larvae: A Comparative Approach, Frontiers in Sustainable Food Systems. (2024) 8, 10.3389/fsufs.2024.1298885, 1298885.

[34] Fox C. J. , Blow P. , Brown J. H. , and Watson I. , The Effect of Various Processing Methods on the Physical and Biochemical Properties of Shrimp Head Meals and Their Utilization by Juvenile, *Penaeus monodon*, Fab, Aquaculture. (1994) 122, no. 2-3, 209–226, 10.1016/0044-8486(94)90511-8.

[35] Reddy V. R. , Reddy V. R. , and Qudratullah S. , Utilisation of Squilla Meal (a Novel Animal Protein Source) by Broilers, British Poultry Science. (1997) 38, no. 3, 263–269, 10.1080/00071669708417984. 9280352

[36] Uczay J. , Battisti E. K. , and Lazzari R. , et al.Fish Meal Replaced by Hydrolysed Soybean Meal in Dietsincreases Growth and Improves the Antioxidant Defensesystem of Silver Catfish (*Rhamdia quelen*), Aquaculture Research. (2019) 50, no. 5, 1438–1447, 10.1111/are.14019.

[37] Feldsine P. , Abeyta C. , Andrews W. H. , and AOAC International Methods Committee , AOAC International Methods Committee Guidelines for Validation of Qualitative and Quantitative Food Microbiological Official Methods of Analysis, Journal of AOAC International. (2002) 85, no. 5, 1187–1200, 10.1093/jaoac/85.5.1187. 12374420

[38] Brett J. R. , Energy Expenditure of Sockeye Salmon, *Oncorhynchus nerka*, During Sustained Performance, Journal of the Fisheries Research Board of Canada. (1973) 30, no. 12, 1799–1809, 10.1139/f73-290.

[39] Kader M. A. , Koshio S. , Ishikawa M. , Yokoyama S. , and Bulbul M. , Supplemental Effects of Some Crude Ingredients in Improving Nutritive Values of Low Fishmeal Diets for Red Seabream, *Pagrus major* , Aquaculture. (2010) 308, no. 3-4, 136–144, 10.1016/j.aquaculture.2010.07.037.

[40] National Research Council (NRC) , Nutrient Requirements of Fish and Shrimp, 2011, National Academies Press.

[41] American Public Health Association (APHA) , Standard Methods for Examination of Water and Wastewater, 2005, 21st edition, American Public Health Association, Standard Methods is a joint publication of the American Public Health Association 101136 10 (APHA), the American Water Works Association (AWWA), and the Water Environment Federation (WEF).

[42] Boyd C. E. , Water Quality in Ponds for Aquaculture, 1990, Alabama Agricultural Experiment Station.

[43] Furné M. , García-Gallego M. , and Hidalgo M. C. , et al.Effect of Starvation and Refeeding on Digestive Enzyme Activities in Sturgeon (*Acipenser naccarii*) and Trout (*Oncorhynchus mykiss*), Comparative Biochemistry and Physiology Part A: Molecular & Integrative Physiology. (2008) 149, no. 4, 420–425, 10.1016/j.cbpa.2008.02.002. 18328757

[44] Bernfeld P. , Amylase α and β, Methods in Enzymology. (1955) 1, 149–158, 10.1016/0076-6879(55)01021-5.

[45] Zamani A. , Hajimoradloo A. , Madani R. , and Farhangi M. , Assessment of Digestive Enzymes Activity During the Fry Development of the Endangered Caspian Brown Trout *Salmo caspius* , Journal of Fish Biology. (2009) 75, no. 4, 932–937, 10.1111/j.1095-8649.2009.02348.x. 20738590

[46] Hummel B. C. W. , A Modified Spectrophotometric Determination of Chymotrypsin, Trypsin, and Thrombin, Canadian Journal of Biochemistry and Physiology. (1959) 37, no. 1, 1393–1399, 10.1139/y59-157. 14405350

[47] Furukawa A. and Tsukahara H. , On the Acid Digestion Method for the Determination of Chromic Oxide as an Index Substance in the Study of Digestibility of Fish Feed, Bulletin of the Japanese Society of Scientific Fisheries. (1966) 32, no. 6, 502–506, 10.2331/suisan.32.502.

[48] Baruah K. , Sahu N. P. , Pal A. K. , Debnath D. , Yengkokpam S. , and Mukherjee S. C. , Interactions of Dietary Microbial Phytase, Citric Acid and Crude Protein Level on Mineral Utilization by Rohu, *Labeo rohita* (Hamilton), Juveniles, Journal of the World Aquaculture Society. (2007) 38, no. 2, 238–249, 10.1111/j.1749-7345.2007.00092.x.

[49] Schneider O. , Amirkolaie A. K. , Vera-Cartas J. , Eding E. H. , Schrama J. W. , and Verreth J. A. J. , Digestibility, Faeces Recovery, and Related Carbon, Nitrogen and Phosphorus Balances of Five Feed Ingredients Evaluated as Fishmeal Alternatives in Nile Tilapia, *Oreochromis niloticus* L., Aquaculture Research. (2004) 35, no. 14, 1370–1379, 10.1111/j.1365-2109.2004.01179.x.

[50] Bancroft J. D. and Gamble M. , Theory and Practice of Histological Technique, 2010, 6th edition, Churchill Livingstone.

[51] Reitman S. and Frankel S. , A Colorimetric Method for the Determination of Serum Glutamic Oxalacetic and Glutamic Pyruvic Transaminases, American Journal of Clinical Pathology. (1957) 28, no. 1, 56–63, 10.1093/ajcp/28.1.56. 13458125

[52] Henry R. J. , Colorimetric Determination of Total Protein., Clinical Chemistry, 1964, Harper and Row, 181.

[53] Wotton I. D. and Freeman H. , Microanalysis in Medical Biochemistry, 1982, Churchill-Livingstone.

[54] Peskin A. V. and Winterbourn C. C. , A Microtiter Plate Assay for Superoxide Dismutase Using a Water-Soluble Tetrazolium Salt (WST-1), Clinica Chimica Acta. (2000) 293, no. 1-2, 157–166, 10.1016/S0009-8981(99)00246-6. 10699430

[55] Ohkawa H. , Ohishi N. , and Yagi K. , Assay for Lipid Peroxides in Animal Tissues by Thiobarbituric Acid Reaction, Analytical Biochemistry. (1979) 95, no. 2, 351–358, 10.1016/0003-2697(79)90738-3. 36810

[56] Beers R. F.Jr. and Sizer I. W. , Spectrophotometric Method for Measuring the Breakdown of Hydrogen Peroxide by Catalase, Journal of Biological Chemistry. (1952) 195, no. 1, 133–140, 10.1016/S0021-9258(19)50881-X. 14938361

[57] Rodriguez-Ariza A. , Toribio F. , and López-Barea J. , Rapid Determination of Glutathione Status in Fish Liver Using High-Performance Liquid Chromatography and Electrochemical Detection, Journal of Chromatography B: Biomedical Sciences and Applications. (1994) 656, no. 2, 311–318, 10.1016/0378-4347(94)00111-1. 7987482

[58] Nagai T. , Inada J. , and Hamada M. , et al.Distribution of Glutathione Peroxidase Activity in Fish, Fisheries Science. (1999) 65, no. 4, 665–666, 10.2331/fishsci.65.665.

[59] SAS , SAS/STAT User Guide, 1996, SAS Institute Inc., Release 6.03 Edition.

[60] Dossou S. , Koshio S. , and Ishikawa M. , et al.Effect of Partial Replacement of Fish Meal by Fermented Rapeseed Meal on Growth, Immune Response and Oxidative Condition of Red Sea Bream Juvenile, *Pagrus major* , Aquaculture. (2018) 490, 228–235, 10.1016/j.aquaculture.2018.02.010.

[61] Yadav K. N. , Deepti M. , and Arun Bhai Patel A. B. , et al.Dissecting Insects as Sustainable Protein Bioresource in Fish Feed for Aquaculture Sustainability, Discover Food. (2025) 5, no. 1, 10.1007/s44187-025-00318-5, 47.

[62] Makkar H. P. S. , Tran G. , Heuzé V. , and Ankers P. , State-of-the-Art on Use of Insects as Animal Feed, Animal Feed Science and Technology. (2014) 197, 1–33, 10.1016/j.anifeedsci.2014.07.008.

[63] Sajid Q. U. A. , Asghar M. U. , Tariq H. , Wilk M. , and Płatek A. , Insect Meal as an Alternative to Protein Concentrates in Poultry Nutrition With Future Perspectives (an Updated Review), Agriculture. (2023) 13, no. 6, 10.3390/agriculture13061239, 1239.

[64] Henry M. , Gasco L. , Piccolo G. , and Fountoulaki E. , Review on the Use of Insects in the Diet of Farmed Fish: Past and Future, Animal Feed Science and Technology. (2015) 203, 1–22, 10.1016/j.anifeedsci.2015.03.001.

[65] Rahman M. and Koh K. , Effects of Formic Acid-Treated Shrimp Meal on Growth Performance and Nutrient Digestibility in Broilers, Journal of Poultry Science. (2016) 53, no. 3, 208–212, 10.2141/jpsa.0160015. PMC7477137 32908385

[66] Rahman M. and Koh K. , Improvement in Nutritional Quality of Shrimp Meal With Autoclave and Chemical Treatments: An In Vitro Study, Journal of Poultry Science. (2016) 53, no. 2, 124–127, 10.2141/jpsa.0150128. PMC7477274 32908374

[67] Ng W.-K. and Koh C.-B. , The Utilization and Mode of Action of Organic Acids in the Feeds of Cultured Aquatic Animals, Reviews in Aquaculture. (2017) 9, no. 4, 342–368, 10.1111/raq.12141.

[68] de la Barca A. M. C. , Ruiz-Salazar R. A. , and Jara-Marini M. E. , Enzymatic Hydrolysis and Synthesis of Soy Protein to Improve Its Amino Acid Composition and Functional Properties, Journal of Food Science. (2000) 65, no. 2, 246–253, 10.1111/j.1365-2621.2000.tb15988.x.

[69] Cândido L. M. B. and Sgarbieri V. C. , Enzymatic Hydrolysis of Nile Tilapia (*Oreochromus Niloticus*) Myofibrillar Proteins: Effects on Nutritional and Hydrophilic Properties, Journal of the Science of Food and Agriculture. (2003) 83, no. 9, 937–944, 10.1002/jsfa.1419.

[70] Foh M. B. K. , Amadou I. , Kamara M. T. , Foh B. M. , and Xia W. , Effect of Enzymatic Hydrolysis on the Nutritional and Functional Properties of Nile Tilapia (*Oreochromis niloticus*) Proteins, American Journal of Biochemistry and Molecular Biology. (2010) 1, no. 1, 54–67, 10.3923/ajbmb.2011.54.67.

[71] Wang C. , Chen S. , and Dai J. , et al.Evaluation of the Enzymatic Hydrolysis of Sargassum Mixed With Fish Protein Hydrolysis Product as an Alternative Protein Source in Shrimp Feed, From the Perspectives of Growth Performance, Amino Acid Composition, Antioxidant Capacity and Endoplasmic Reticulum Stress, Aquaculture Reports. (2025) 42, 10.1016/j.aqrep.2025.102813, 102813.

[72] Mamauag R. , Koshio S. , and Ishikawa M. , et al.Soy Peptide Inclusion Levels Influence the Growth Performance, Proteolytic Enzyme Activities, Blood Biochemical Parameters and Body Composition of Japanese Flounder, *Paralichthys olivaceus* , Aquaculture. (2011) 321, no. 3-4, 252–258, 10.1016/j.aquaculture.2011.09.022.

[73] Terrey D. , James J. , and Tankovski I. , et al.Palatability Enhancement Potential of, *Hermetia illucens*, Larvae Protein Hydrolysate in, *Litopenaeus vannamei*, Diets, Molecules. (2021) 26, no. 6, 10.3390/molecules26061582, 1582. 33805599 PMC8002068

[74] Kroeckel S. , Harjes A.-G. , and Roth I. , et al.When a Turbot Catches a Fly: Evaluation of a Pre-Pupae Meal of the Black Soldier Fly (*Hermetia illucens*) as Fish Meal Substitute—Growth Performance and Chitin Degradation in Juvenile Turbot (*Psetta maxima*), Aquaculture. (2012) 364-365, 345–352, 10.1016/j.aquaculture.2012.08.041.

[75] Belghit I. , Liland N. S. , and Waagbø R. , et al.Potential of Insect-Based Diets for Atlantic Salmon (*Salmo salar*), Aquaculture. (2018) 491, 72–81, 10.1016/j.aquaculture.2018.03.016.

[76] Palupi E. T. , Setiawati M. , Lumlertdacha S. , and Suprayudi M. A. , Growth Performance, Digestibility, and Blood Biochemical Parameters of Nile Tilapia (*Oreochromis niloticus*) Reared in Floating Cages and Fed Poultry By-Product Meal, Journal of Applied Aquaculture. (2020) 32, no. 1, 16–33, 10.1080/10454438.2019.1605324.

[77] Guerreiro I. , Serra C. R. , and Coutinho F. , et al.Digestive Enzyme Activity and Nutrient Digestibility in Meagre (*Argyrosomus regius*) Fed Increasing Levels of Black Soldier Fly Meal (*Hermetia illucens*), Aquaculture Nutrition. (2021) 27, no. 1, 142–152, 10.1111/anu.13172.

[78] Muin H. and Taufek N. M. , Evaluation of Growth Performance, Feed Efficiency and Nutrient Digestibility of Red Hybrid Tilapia Fed Dietary Inclusion of Black Soldier Fly Larvae (*Hermetia illucens*), Aquaculture and Fisheries. (2024) 9, no. 1, 46–51, 10.1016/j.aaf.2022.09.006.

[79] Mahmoud R. E. , Gadallah H. , and Orma O. A. , Effects of Replacing Protein of Fishmeal With Protein of Poultry By-Product Meal on Growth Performance, Body Composition, Liver Histological Changes and Selected Serum Parameters of Nile Tilapia, Journal of Advanced Veterinary Research. (2023) 13, no. 6, 871–876.

[80] Tippayadara N. , Dawood M. A. O. , Krutmuang P. , Hoseinifar S. H. , Doan H. V. , and Paolucci M. , Replacement of Fish Meal by Black Soldier Fly (*Hermetia illucens*) Larvae Meal: Effects on Growth, Haematology, and Skin Mucus Immunity of Nile Tilapia, *Oreochromis niloticus* , Animals. (2021) 11, no. 1, 10.3390/ani11010193, 193. 33467482 PMC7830215

[81] Perez-Velazquez M. , Millanes-Mora M. A. , and González-Félix M. L. , Assessment of Hydrolysis of Partially Defatted Black Soldier Fly Larvae Meal in Diets for Nile Tilapia *Oreochromis niloticus* , Animal Feed Science and Technology. (2024) 307, 10.1016/j.anifeedsci.2023.115831, 115831.

[82] Yi C. , Huang D. , Yu H. , Gu J. , Liang H. , and Ren M. , Enzymatically Hydrolyzed Poultry By-Product Supplementation, Instead of Fishmeal, Alone Improves the Quality of Largemouth Bass (*Micropterus salmoides*) Back Muscle Without Compromising Growth, Foods. (2023) 12, no. 18, 10.3390/foods12183485, 3485. 37761194 PMC10529141

[83] Kureshy N. , Davis D. A. , and Arnold C. R. , Partial Replacement of Fish Meal With Meat-and-Bone Meal, Flash-Dried Poultry By-Product Meal, and Enzyme-Digested Poultry By-Product Meal in Practical Diets for Juvenile Red Drum, North American Journal of Aquaculture. (2000) 62, no. 4, 266–272.

[84] Prandi B. , Samaei S. , Beninati F. , Nardi A. , Tedeschi T. , and Sforza S. , Exploitation of Bones-Rich Poultry By-Products to Produce Protein Hydrolysates: Optimization of Hydrolysis Parameters and Chemical Characterization, Poultry Science. (2024) 103, no. 8, 10.1016/j.psj.2024.103924, 103924. PMC11253654 38908125

[85] Zhao J. , Liu Y. , and Jiang J. , et al.Effects of Dietary Isoleucine on Growth, the Digestion and Absorption Capacity and Gene Expression in Hepatopancreas and Intestine of Juvenile Jian Carp (*Cyprinus carpio*, var. Jian), Aquaculture. (2012) 368-369, 117–128, 10.1016/j.aquaculture.2012.09.019.

[86] Shimizu M. , Food-Derived Peptides and Intestinal Functions, BioFactors. (2004) 21, no. 1–4, 43–47, 10.1002/biof.552210109. 15630168

[87] Santos J. F. , Castro P. F. , and Gonçalves-Leal A. L. , et al.Digestive Enzyme Activity in Juvenile Nile Tilapia (*Oreochromis niloticus*, L) Submitted to Different Dietary Levels of Shrimp Protein Hydrolysate, Aquaculture International. (2013) 21, 563–577.

[88] Nobile V. , Duclos E. , Michelotti A. , Bizzaro G. , Negro M. , and Soisson F. , Supplementation With a Fish Protein Hydrolysate (*Micromesistius poutassou*): Effects on Body Weight, Body Composition, and CCK/GLP-1 Secretion, Food & Nutrition Research. (2016) 60, no. 1, 10.3402/fnr.v60.29857, 29857. 26829186 PMC4734037

[89] Song Z. , Li P. , Wang J. , Sun Y. , and Wang C. , Dietary Inclusion of Hydrolyzed Soybean and Cottonseed Meals Influence Digestion, Metabolic Enzymes, and Growth-Related Hormones and Growth of Juvenile Turbot (*Scophthalmus maximus*), Aquaculture International. (2018) 26, no. 4, 1017–1033, 10.1007/s10499-018-0265-z.

[90] Dawood M. A. O. , Magouz F. I. , Essa M. , and Mansour M. , Impact of Yeast Fermented Poultry By-Product Meal on Growtһ, Digestive Enzyme Activities, Intestinal Morpһometry and Immune Response Traits of Common Carp (*Cyprinus carpio*), Annals of Animal Science. (2020) 20, no. 3, 939–959, 10.2478/aoas-2020-0021.

[91] Dawood M. A. O. , Magouz F. I. , and Mansour M. , et al.Evaluation of Yeast Fermented Poultry By-Product Meal in Nile Tilapia (*Oreochromis niloticus*) Feed: Effects on Growth Performance, Digestive Enzymes Activity, Innate Immunity, and Antioxidant Capacity, Frontiers in Veterinary Science. (2020) 6, 10.3389/fvets.2019.00516, 516. 32047756 PMC6996487

[92] Rønnestad I. , Yúfera M. , Ueberschär B. , Ribeiro L. , Sæle Ø. , and Boglione C. , Feeding Behaviour and Digestive Physiology in Larval Fish: Current Knowledge, and Gaps and Bottlenecks in Research, Reviews in Aquaculture. (2013) 5, no. s1, S59–S98, 10.1111/raq.12010.

[93] Khempaka S. , Mochizuki M. , Koh K. , and Karasawa Y. , Effect of Chitin in Shrimp Meal on Growth Performance and Digestibility in Growing Broilers, The Journal of Poultry Science. (2006) 43, no. 4, 339–343, 10.2141/jpsa.43.339.

[94] Kim W. K. and Patterson P. H. , Nutritional Value of Enzyme-or Sodium Hydroxide-Treated Feathers From Dead Hens, Poultry Science. (2000) 79, no. 4, 528–534, 10.1093/ps/79.4.528. 10780649

[95] Soliman M. A. M. , El-Kholy K. F. , El-helaly M. A. E.-f. , and Soluma A. , Effect of Replacing Soybean Meal by Slaughterhouse Poultry By-Products Meal on Nutrient Digestibility Ecoefficiency and Accumulation of Elements of the Nile Tilapia (*Oreochromis niloticus* L.) Under Aquaponics System Conditions, Egyptian Journal of Aquatic Biology and Fisheries. (2022) 26, no. 6, 719–736, 10.21608/ejabf.2022.275466.

[96] Reda R. M. , Mahmoud R. , Selim K. M. , and El-Araby I. E. , Effects of Dietary Acidifiers on Growth, Hematology, Immune Response and Disease Resistance of Nile Tilapia, *Oreochromis niloticus* , Fish & Shellfish Immunology. (2016) 50, 255–262, 10.1016/j.fsi.2016.01.040. 26860238

[97] Vielma J. and Lall S. , Dietary Formic Acid Enhances Apparent Digestibility of Minerals in Rainbow Trout, *Oncorhynchus mykiss* (Walbaum), Aquaculture Nutrition. (1997) 3, no. 4, 265–268.

[98] Takakuwa F. , Tanabe R. , and Nomura S. , et al.Availability of Black Soldier Fly Meal as an Alternative Protein Source to Fish Meal in Red Sea Bream (*Pagrus major*, Temminck & Schlegel) Fingerling Diets, Aquaculture Research. (2022) 53, no. 1, 36–49, 10.1111/are.15550.

[99] Fontes T. V. , de Oliveira K. R. B. , and Gomes Almeida I. L. , et al.Digestibility of Insect Meals for Nile Tilapia Fingerlings, Animals. (2019) 9, no. 4, 10.3390/ani9040181, 181. 31010009 PMC6523303

[100] Dumas A. , Raggi T. , Barkhouse J. , Lewis E. , and Weltzien E. , The Oil Fraction and Partially Defatted Meal of Black Soldier Fly Larvae (*Hermetia illucens*) Affect Differently Growth Performance, Feed Efficiency, Nutrient Deposition, Blood Glucose and Lipid Digestibility of Rainbow Trout (*Oncorhynchus mykiss*), Aquaculture. (2018) 492, 24–34, 10.1016/j.aquaculture.2018.03.038.

[101] Gasco L. , Biasato I. , Dabbou S. , Schiavone A. , and Gai F. , Animals Fed Insect-Based Diets: State-of-the-Art on Digestibility, Performance and Product Quality, Animals. (2019) 9, no. 4, 10.3390/ani9040170, 170. 30995783 PMC6523619

[102] Esteban M. A. , Mulero V. , Cuesta A. , Ortuño J. , and Meseguer J. , Effects of Injecting Chitin Particles on the Innate Immune Response of Gilthead Seabream (*Sparus aurata* L.), Fish & Shellfish Immunology. (2000) 10, no. 6, 543–554, 10.1006/fsim.2000.0271. 11016588

[103] Gopalakannan A. and Arul V. , Immunomodulatory Effects of Dietary Intake of Chitin, Chitosan and Levamisole on the Immune System of *Cyprinus carpio* and Control of *Aeromonas hydrophila* Infection in Ponds, Aquaculture. (2006) 255, no. 1–4, 179–187, 10.1016/j.aquaculture.2006.01.012.

[104] Lock E.-J. , Biancarosa I. , and Gasco L. , Halloran A. , Flore R. , Vantomme P. , and Roos N. , Insects as Raw Materials in Compound Feed for Aquaculture, Edible Insects in Sustainable Food Systems, 2018, Springer, 10.1007/978-3-319-74011-9_16.

[105] Poolsawat L. , Yang H. , Sun Y.-F. , Li X.-Q. , Liang G.-Y. , and Leng X.-J. , Effect of Replacing Fish Meal With Enzymatic Feather Meal on Growth and Feed Utilization of Tilapia (*Oreochromis niloticus* × *O. aureus*), Animal Feed Science and Technology. (2021) 274, 10.1016/j.anifeedsci.2021.114895, 114895.

[106] Mendoza R. , De Dios A. , and Vazquez C. , et al.Fishmeal Replacement With Feather-Enzymatic Hydrolyzates Co-Extruded With Soya-Bean Meal in Practical Diets for the Pacific White Shrimp (*Litopenaeus vannamei*), Aquaculture Nutrition. (2001) 7, no. 3, 143–151, 10.1046/j.1365-2095.2001.00164.x.

[107] Bieczynski F. , Painefilú J. C. , Venturino A. , and Luquet C. M. , Expression and Function of ABC Proteins in Fish Intestine, Frontiers in Physiology. (2021) 12, 10.3389/fphys.2021.791834, 791834. 34955897 PMC8696203

[108] Jutfelt F. , Farrell A. P. , Barrier Function of the Gut, Encyclopedia of Fish Physiology: From Genome to Environment, 2011, 2, 1322–1331.

[109] Dimitroglou A. , Merrifield D. L. , and Moate R. , et al.Dietary Mannan Oligosaccharide Supplementation Modulates Intestinal Microbial Ecology and Improves Gut Morphology of Rainbow Trout, *Oncorhynchus mykiss* (Walbaum), Journal of Animal Science. (2009) 87, no. 10, 3226–3234, 10.2527/jas.2008-1428. 19617514

[110] Hershberg R. and Blumberg R. S. , Targan S. R. , Shanahan F. , and Karp L. C. , The Lymphocyte-Epithelial-Bacterial Interface, Inflammatory Bowel Disease: From Bench to Bedside, 2003, Springer, 121–146, 10.1007/0-387-25808-6_6.

[111] Kim Y. S. and Ho S. B. , Intestinal Goblet Cells and Mucins in Health and Disease: Recent Insights and Progress, Current Gastroenterology Reports. (2010) 12, no. 5, 319–330, 10.1007/s11894-010-0131-2. 20703838 PMC2933006

[112] Yao W. , Zhang C. , and Mao H. , et al.Effects of Dietary Defatted Black Soldier Fly (*Hermetia illucens*) Larvae Meal Substituting Fish Meal on Growth, Antioxidative Capacity, Immunity, Intestinal Histology and Microbiota of Juvenile Chinese Mitten Crab (*Eriocheir sinensis*), Aquaculture Reports. (2024) 38, 10.1016/j.aqrep.2024.102302, 102302.

[113] Xu X. , Ji H. , Yu H. , and Zhou J. , Influence of Dietary Black Soldier Fly (*Hermetia illucens* Linnaeus) Pulp on Growth Performance, Antioxidant Capacity and Intestinal Health of Juvenile Mirror Carp (*Cyprinus carpio* var. Specularis), Aquaculture Nutrition. (2019) 26, no. 2, 432–443, 10.1111/anu.13005.

[114] Harikrishnan R. , Balasundaram C. , and Heo M.-S. , Effect of *Inonotus obliquus* Enriched Diet on Hematology, Immune Response, and Disease Protection in Kelp Grouper, *Epinephelus bruneus* Against *Vibrio harveyi* , Aquaculture. (2012) 344-349, 48–53, 10.1016/j.aquaculture.2012.03.010.

[115] Yu P. , Chen H. , and Liu M. , et al.Current Status and Application of Largemouth Bass (*Micropterus salmoides*) Germplasm Resources, Reproduction and Breeding. (2024) 4, no. 2, 73–82, 10.1016/j.repbre.2024.01.004.

[116] Zhou J. S. , Liu S. S. , Ji H. , and Yu H. B. , Effect of Replacing Dietary Fish Meal With Black Soldier Fly Larvae Meal on Growth and Fatty Acid Composition of Jian Carp (*Cyprinus carpio* var. Jian), Aquaculture Nutrition. (2018) 24, no. 1, 424–433, 10.1111/anu.12574.

[117] Yildirim-Aksoy M. , Eljack R. , Schrimsher C. , and Beck B. H. , Use of Dietary Frass From Black Soldier Fly Larvae, *Hermetia illucens*, in Hybrid Tilapia (Nile × Mozambique, *Oreocromis niloticus* × *O. mozambique*) Diets Improves Growth and Resistance to Bacterial Diseases, Aquaculture Reports. (2020) 17, 10.1016/j.aqrep.2020.100373, 100373.

[118] Eid A.-H. , Hashem M. S. , Ibrahem A. A. , Ali B. A. , and Badawy L. A. , Growth and Physiological Response of the Nile Tilapia (*Oreochromis niloticus*) Fed a Fermented Poultry By-Product Meal, Egyptian Journal of Aquatic Biology and Fisheries. (2024) 28, no. 4, 1023–1038, 10.21608/ejabf.2024.370892.

[119] Sathishkumar G. , Felix N. , and Prabu E. , Effects of Dietary Protein Substitution of Fish Meal With Bioprocessed Poultry By-Product Meal on Growth Performances, Nutrient Utilization, Whole-Body Composition and Haemato-Biochemical Responses of GIFT Tilapia Reared in Floating Cages, Aquaculture Research. (2021) 52, no. 11, 5407–5418, 10.1111/are.15410.

[120] Abdel-Tawwab M. , Khalil R. H. , Metwally A. A. , Shakweer M. S. , Khallaf M. A. , and Abdel-Latif H. M. R. , Effects of Black Soldier Fly (*Hermetia illucens* L.) Larvae Meal on Growth Performance, Organs-Somatic Indices, Body Composition, and Hemato-Biochemical Variables of European Sea Bass, *Dicentrarchus labrax* , Aquaculture. (2020) 522, 10.1016/j.aquaculture.2020.735136, 735136.

[121] Zhang C. , Wang N. , Xu Y. , Tan H.-Y. , Li S. , and Feng Y. , Molecular Mechanisms Involved in Oxidative Stress-Associated Liver Injury Induced by Chinese Herbal Medicine: An Experimental Evidence-Based Literature Review and Network Pharmacology Study, International Journal of Molecular Sciences. (2018) 19, no. 9, 10.3390/ijms19092745, 2745. 30217028 PMC6165031

[122] Lei X. G. , Zhu J. H. , and Cheng W. H. , et al.Paradoxical Roles of Antioxidant Enzymes: Basic Mechanisms and Health Implications, Physiological Reviews. (2016) 96, 307–364. 26681794 10.1152/physrev.00010.2014 PMC4839492

